# NSAID use as a shared decision-making variable in musculoskeletal rehabilitation and strength and conditioning: a perspective

**DOI:** 10.3389/fspor.2026.1912725

**Published:** 2026-09-30

**Authors:** Longji Li, Haojie Long, Lifeng Zhang

**Affiliations:** Strength and Conditioning Center, School of Physical Education, Chengdu Sport University, Chengdu, China

**Keywords:** load management, musculoskeletal rehabilitation, nonsteroidal anti-inflammatory drugs, shared decision-making, symptom interpretation

## Abstract

Pain relief is often considered a key indicator of musculoskeletal rehabilitation and load progression in physical training. However, nonsteroidal anti-inflammatory drugs (NSAIDs) can alter pain perception and symptom expression, posing new interpretive challenges to symptom-based clinical judgment. During NSAID use, decreased pain and improved training completion do not necessarily indicate a synchronous recovery of tissue load tolerance. Adjusting load solely based on immediate symptom changes may lead to an overestimation of rehabilitation progress. This article explores the potential impact of NSAID use on rehabilitation decisions from the perspectives of symptom interpretation and load management. Pharmacological status should be considered a crucial variable influencing clinical reasoning, rather than merely background information in the medical history. Based on the shared decision-making principle, this article proposes the DICE framework (Document, Interpret, Coordinate, Educate), which integrates NSAID-related information into rehabilitation and load-management decisions by standardizing medication information recording, interpreting symptom changes in conjunction with medication context, promoting multidisciplinary team collaboration, and strengthening medication education. This framework is intended to support more consistent symptom interpretation, more informed load progression decisions, and improved communication within rehabilitation teams. This article argues that NSAID use should be incorporated into a dynamic assessment system for rehabilitation and training decisions to more accurately differentiate changes in symptom control, functional recovery, and load tolerance, thereby supporting safer, more transparent, and patient-centered rehabilitation practices.

## 1 Introduction

In musculoskeletal rehabilitation and strength and conditioning, pain reduction is commonly regarded as a positive clinical signal (1). When patients, athletes, or individuals undergoing rehabilitation report reduced pain and improved training completion, physical therapists and strength and conditioning professionals may consider increasing training intensity, training volume, or movement complexity (2). However, when symptomatic improvement occurs during NSAID use, medication status may influence the interpretation of recovery-related signals (3). The key clinical challenge is therefore how NSAID-related information can be incorporated into shared decision-making regarding rehabilitation progression.

NSAIDs are widely used to manage acute and chronic musculoskeletal pain, sport-related injuries, and inflammation-related symptoms (4). Guidelines for acute musculoskeletal injury pain management, consensus statements on pain management in elite athletes, and best-practice recommendations for musculoskeletal pain indicate that pain management is not merely a matter of selecting a single medication (5, 6). Rather, it should be understood within the context of patient goals, functional recovery, risk communication, and interprofessional collaboration (7). In sport and rehabilitation settings, pain control is often intertwined with training participation, functional recovery, and return-to-activity goals (8). Therefore, NSAID use has direct practical relevance.

A current practice gap is that NSAID use is often documented as part of the medical history or medication profile but is not consistently incorporated into clinical reasoning (9). When developing progression plans, physical therapists and strength and conditioning professionals typically consider pain scores, post-activity responses, swelling, range of motion, strength, movement quality, and functional test performance (10). However, NSAIDs may alter pain perception and inflammation-related symptoms, making patient-reported immediate symptom responses more difficult to interpret (11, 12). Lower pain scores remain clinically meaningful. In the context of medication use, they should not be equated in isolation with greater tissue load tolerance (13, 14).

Therefore, this article argues that NSAID use should not be regarded merely as background medication information (15). Rather, it should be considered a practical variable in shared decision-making within musculoskeletal rehabilitation and strength and conditioning (16, 17). The purpose is not to restrict patient participation in rehabilitation or training or to make a simplistic judgment about NSAID use. Instead, the aim is to make relevant medication information visible within team-based reasoning. When physical therapists, strength and conditioning professionals, physicians, and patients share a common understanding of NSAID use, they can interpret symptom changes more cautiously, establish load-progression criteria more consistently, and distinguish more clearly between short-term symptom control and actual functional recovery. Although the principles of DICE may be applicable across musculoskeletal rehabilitation settings, this framework is particularly relevant to physically active individuals and athletes, where symptom interpretation is closely linked with training progression, return-to-performance decisions, and sport-specific demands.

## 2 Article type

Perspective.

## 3 From pain control to symptom interpretation: what does NSAID use change?

In musculoskeletal rehabilitation and strength and conditioning, progression decisions typically do not rely on a single indicator (18–20). Instead, they are based on an integrated assessment of symptoms, function, and load responses (21). Physical therapists and strength and conditioning professionals consider discomfort during exercise, responses several hours after training, next-day pain or stiffness, local swelling, range of motion, strength performance, movement quality, and functional test results (22, 23). Pain-monitoring models and research on tendinopathy rehabilitation also suggest that symptom responses can inform activity modification and load progression (24, 25). However, their value lies in supporting overall load management rather than serving as an isolated criterion for progression (26, 27). Studies on Achilles tendinopathy and subsequent return-to-sport protocols emphasize that symptoms, function, and recovery time should be considered together when evaluating rehabilitation progress (28, 29).

NSAID use has practical significance. NSAIDs exert their pharmacological effects primarily by inhibiting cyclooxygenase (COX) enzymes, including constitutively expressed COX-1 and inducible COX-2. These enzymes catalyze the conversion of arachidonic acid into prostaglandins and prostacyclin, lipid mediators that regulate inflammation, vascular tone, fever, and pain sensitization. During tissue injury or inflammation, COX-2 expression is upregulated, leading to increased prostaglandin production, which promotes vasodilation, vascular permeability, leukocyte recruitment, and sensitization of peripheral nociceptors. By reducing prostaglandin synthesis, NSAIDs dampen inflammatory signaling, decrease nociceptor excitability, reduce central pain amplification, and thereby produce analgesic, anti-inflammatory, and antipyretic effects (30). These analgesic and anti-inflammatory effects can modify symptom expression during rehabilitation and training, making observed changes in pain or discomfort difficult to interpret in isolation. Therefore, symptomatic improvement during NSAID use may reflect medication-related symptom control, true recovery, training adaptation, load modification, or a combination of these factors (31, 32). These different interpretations may lead to different implications for load progression decisions.

Beyond symptom modulation, NSAIDs may influence physiological processes involved in musculoskeletal adaptation and tissue repair through inhibition of COX activity and subsequent reduction in prostaglandin synthesis. In skeletal muscle, a randomized controlled trial reported greater increases in quadriceps muscle cross-sectional area and volume after 12 weeks of resistance training combined with diclofenac, accompanied by distinct changes in muscle gene expression but no additional gains in strength, suggesting that NSAIDs can alter the pattern of skeletal muscle adaptation rather than simply suppress it (33). In clinical studies of rotator cuff repair, current evidence does not indicate a significant adverse effect of postoperative NSAID use on retear rates. A systematic review and meta-analysis of seven randomized controlled trials involving 507 patients found no significant difference in retear rates after arthroscopic rotator cuff repair with postoperative NSAID use (34). In bone healing, prostaglandin signaling is involved in osteoblast and osteoclast activity and in the inflammatory and reparative responses following skeletal injury (35). Although these mechanisms provide a biological basis for NSAID-related modulation of bone repair, a meta-analysis including 20 studies and 523,240 patients found no statistically significant association between NSAID use and delayed union or non-union after adjustment for confounding factors (36). In a murine tibial-fracture model, NSAID administration during the active phase of the circadian cycle improved postoperative recovery, whereas administration during the resting phase impaired bone healing (37). This animal study suggests that the timing of NSAID administration may influence postoperative recovery and bone healing. However, whether this timing-dependent effect is clinically relevant to humans remains uncertain.

Overall, clinical evidence remains heterogeneous. Therefore, NSAID use should be interpreted as one contextual factor within rehabilitation decision-making rather than as an independent predictor of tissue healing or readiness for load progression.

## 4 Why does NSAID use influence training decisions?

Load progression in rehabilitation and strength and conditioning should not be viewed as an immediate reward for a single low-symptom training session. Rather, it should be understood as an ongoing process of calibration (38, 39). Training load includes not only weight or intensity but also volume, frequency, movement complexity, speed, and sport-specific demands (40). Load tolerance refers to the capacity of an individual to tolerate progressively increased physical demands without unacceptable symptom exacerbation, functional deterioration, or impaired recovery responses. Return-to-sport consensus statements emphasize that rehabilitation progress and readiness to progress or return to activity should be based on multidimensional judgment across a continuum, rather than on a single test or training response (41). Research on training load also indicates that the rate of load change, individual load capacity, and recovery status collectively influence adaptation and injury risk (42). Therefore, progression decisions should integrate immediate performance, delayed symptoms, functional output, and recovery quality.

During NSAID use, load progression decisions may become more complex because symptom responses are interpreted within a modified pharmacological context. Current evidence does not support a uniform interpretation that all NSAID use adversely affects rehabilitation outcomes, particularly when short-term, clinically indicated use is considered. In patients, athletes, or individuals undergoing rehabilitation, reduced discomfort during training, improved subjective tolerance, or better training completion may suggest that the current load is acceptable. However, these changes may also partly reflect medication-related symptom control. In particular, load tolerance may be overestimated when progression decisions rely primarily on immediate sensations during exercise or training completion (43).

NSAID use should not automatically determine whether progression occurs. Rather, it should modify how the team weighs progression-related signals (44). During medication use, the team should place greater emphasis on responses several hours after training and the following day, local swelling or stiffness, range of motion, strength performance, movement quality, and recent changes in load (45). In rehabilitation contexts requiring staged load exposure, progression criteria should integrate symptom responses, functional capacity, and recovery time rather than rely solely on patient reports of feeling better or completing the training session (46).

## 5 Shared decision-making: making NSAID use shared information within the team

When NSAID use is not shared within the team, the same symptomatic improvement may be interpreted differently by different stakeholders (47). Physicians may focus on medication efficacy and safety, physical therapists on tissue responses and functional recovery, strength and conditioning professionals on training completion and readiness for progression, and patients, athletes, or individuals undergoing rehabilitation may interpret the improvement as recovery. Such differences in interpretation do not necessarily reflect errors in professional judgment. Rather, they may arise from a failure to use key information collectively. Therefore, shared decision-making provides a framework for integrating these perspectives by ensuring that medication status, rehabilitation responses, and individual goals are considered collectively rather than interpreted separately by different stakeholders.

Shared decision-making involves more than interprofessional coordination and information sharing. It requires that patients, athletes, or individuals undergoing rehabilitation actively participate in decisions regarding rehabilitation goals, acceptable risk, available management options, and the interpretation of uncertainty associated with recovery during NSAID use. It means that each role uses the same key information within its own professional scope (48). Physicians or prescribers are responsible for NSAID indications, safety, contraindications, and medication adjustments. Physical therapists interpret symptom irritability, tissue responses, and functional recovery. Strength and conditioning professionals manage training exposure, movement quality, and sport-specific progression. Patients, athletes, or individuals undergoing rehabilitation should accurately report medication use, symptom changes, responses to physical loading, and personal goals relevant to rehabilitation progression. Integrating these perspectives allows the team to discuss not only clinical findings but also individual preferences, expected benefits, potential risks, and acceptable levels of uncertainty when determining rehabilitation progression. Medication-related decisions should therefore reflect not only clinical judgment but also individual preferences and values.

This approach is consistent with the principles of patient-centered care, information sharing, and team-based collaboration emphasized in best-practice recommendations for musculoskeletal pain and consensus statements on pain management in athletes (49). High-quality rehabilitation decisions depend not only on individual examination findings or isolated training performance, but also on whether the team can develop a consistent interpretation based on the same set of clinical information.

Regarding NSAID use, the key question is not merely whether the patient is currently taking medication, but how current medication use affects the interpretation of symptoms, function, and load responses. The purpose of incorporating NSAID use into shared decision-making is therefore to improve transparency regarding the factors influencing rehabilitation decisions, rather than to create additional medication-management responsibilities for non-prescribing professionals. In sport settings, shared decision-making may be particularly challenging because athletes are often influenced by external pressures, including competition schedules, coaching expectations, contractual obligations, and team performance demands. These competing priorities may encourage premature progression despite incomplete recovery. It is crucial to communicate uncertainties openly and discuss potential risks clearly to ensure that rehabilitation decisions are based on both medical evidence and the athlete's long-term interests. The team needs a simple, repeatable process that translates medication use into practical information for symptom interpretation, load progression, and patient education.

## 6 From concept to practice: the DICE framework

Existing pain-monitoring models, load-management approaches, and return-to-sport frameworks provide important guidance for rehabilitation progression by incorporating symptom responses, functional capacity, and training responses into decision-making. Shared decision-making frameworks emphasize collaborative communication, patient preferences, and multidisciplinary participation throughout the rehabilitation process. However, these approaches generally provide limited operational guidance regarding how concurrent medication use should be incorporated into symptom interpretation and load-management decisions. Similarly, although shared decision-making frameworks emphasize communication, patient involvement, and collaborative care, medication status is not consistently integrated as an explicit variable within rehabilitation reasoning. Therefore, NSAID use may remain documented as part of medical history without being systematically considered when interpreting symptoms or determining progression.

Based on this practical need, this article proposes the DICE framework (Document, Interpret, Coordinate, and Educate), which aims to transform NSAID use status from passive medical-record information into shared information that can be incorporated into clinical reasoning. The value of DICE does not lie in introducing new clinical principles, but in providing a structured approach to integrate NSAID-related information into existing rehabilitation decision-making processes. Rather than replacing established pain-monitoring, load-management, return-to-sport, or shared decision-making frameworks, DICE is proposed as a medication-aware reasoning layer that complements these existing approaches. It is intended to support more consistent interpretation of symptoms, facilitate communication among rehabilitation stakeholders, and inform decisions regarding load progression by ensuring that medication context is considered alongside functional and clinical information. The purpose of this framework is not to replace medication-prescribing guidelines (50) or require physical therapists or strength and conditioning professionals to assume medication-management responsibilities. Instead, it serves as a medication-aware layer that facilitates the interpretation of symptom changes across different professional roles within an appropriate pharmacological context and supports more informed load progression and patient communication. When applied in practice, DICE encourages rehabilitation teams to consider NSAID status alongside symptom responses, functional capacity, and load tolerance before making decisions regarding load progression and return-to-activity or return-to-performance progression, depending on the individual's rehabilitation goals and context (Table 1). In this framework, return to activity refers to resumption of desired daily or recreational activities, whereas return to performance refers to restoration of sport-specific capacities required for competitive participation.

**Table 1:** Illustrative conceptual application of the DICE framework across selected musculoskeletal rehabilitation scenarios.

Context	Key contextual modifiers	Main adaptation of DICE	Primary caution	Load progression considerations
Acute soft-tissue injury	Tissue involved; injury severity; NSAID timing	Emphasize delayed swelling, range of motion, and next-day response	Pain relief may mask early irritability	Consider avoiding progression based solely on low pain
Chronic tendinopathy	Symptom duration; cumulative loading; NSAID duration	Emphasize 24-hour response and weekly load trends	Low pain may not equal tendon capacity	Consider progression after repeated tolerance
Bone injury	Fracture type; healing stage; imaging findings	Use more conservative symptom interpretation	Pain may be a warning signal	Consider load progression only after healing criteria are satisfied
Postoperative rehabilitation	Surgical procedure; healing phase; prescribed NSAID regimen	Integrate surgical milestones and objective tests	Pain relief may not reflect tissue integration	Follow stage-specific progression in conjunction with objective assessment
Late-stage return to sport	Competition demands; medication timing; repeated testing	Document medication use during testing	Medicated performance may overestimate readiness	Confirm stable performance across repeated exposures

Footnote: Progression implications are illustrative conceptual examples intended to demonstrate medication-aware clinical reasoning within the DICE framework and should not be interpreted as empirically validated progression criteria. Clinical decisions should be individualized according to tissue type, healing stage, NSAID characteristics (class, dose, timing, duration), and patient-specific factors.

Unlike existing rehabilitation frameworks, which primarily focus on symptom progression and functional performance, DICE begins with systematic documentation of medication context because subsequent interpretation depends on whether symptom responses occur under pharmacological modulation. First, document. NSAID use should not be recorded merely as “currently using” or “not using.” A more informative documentation approach may capture the medication context that could influence subsequent symptom interpretation, including the indication for use, NSAID type, dosage, frequency, duration, timing relative to rehabilitation or training sessions, and whether the patient has independently initiated, discontinued, or modified medication use. Importantly, documenting the temporal relationship between medication administration and physical loading may provide additional clinical context. For example, NSAID use before training to ensure participation may have different implications for symptom interpretation compared with NSAID use after training to manage residual discomfort (51). By incorporating these details into routine rehabilitation communication, teams can better interpret whether symptom changes reflect medication-related symptom control, changes in tissue response, or broader recovery processes, while also supporting physicians or prescribers in evaluating medication indications, safety considerations, and potential risks.

Second, interpret. During NSAID use, pain scores and symptom responses remain clinically valuable, but they should be interpreted within the context of medication use. Symptom responses should be evaluated alongside medication timing, delayed responses to loading, and objective indicators of functional recovery. Therefore, symptom changes should be evaluated within a broader pattern of recovery and load tolerance, including symptom responses several hours after loading, next-day pain or stiffness, swelling, range of motion, strength performance, movement quality, and functional test responses. For example, an individual who reports minimal pain during exercise may appear ready for progression; however, if delayed symptoms increase after training or functional performance declines when the medication effect decreases, the current load may not yet be appropriate for progression.

Third, coordinate. NSAID use should become shared information in interprofessional communication. Physicians or prescribers focus on indications, dosage, contraindications, and safety. Physical therapists focus on symptom irritability, tissue responses, and functional recovery. Strength and conditioning professionals focus on training load, movement quality, and sport performance. Patients, athletes, or individuals undergoing rehabilitation provide information on pain experience, post-training responses, and goal preferences. For example, an athlete may experience reduced pain, improved training participation, and better movement quality during exercise after NSAID use; however, increased stiffness, recurrent symptoms, or impaired functional performance may occur on the following day. Sharing these observations within the rehabilitation team may help differentiate medication-related symptom relief from meaningful recovery progression. Physical therapists, strength and conditioning professionals, and prescribing clinicians can then integrate functional assessments, training responses, and medication-related considerations to inform decisions regarding whether the current load should be progressed, maintained, or modified. The goal of coordination is not to blur professional boundaries or transfer medication-management responsibilities to non-prescribing professionals. Rather, it is to ensure that different team members do not interpret the same symptom change based on incomplete or inconsistent information.

Fourth, educate. Patients should understand that NSAID-related symptom relief does not necessarily indicate full recovery of load tolerance. The goal of education is not to create fear of NSAIDs. Rather, it is to help patients distinguish among pain control, functional improvement, and rehabilitation readiness. Patients should also be encouraged to accurately report medication timing, post-training responses, and any adverse symptoms so that the team can adjust rehabilitation and training plans promptly.

Through the DICE framework, NSAID use can shift from background medication information to a dynamic variable in rehabilitation reasoning. Rather than allowing symptom improvement during NSAID use to independently determine progression decisions, DICE encourages teams to integrate medication status with symptom responses, functional capacity, load tolerance, patient preferences, and safety considerations when deciding whether to advance rehabilitation stages, increase training loads, or progress return-to-performance activities. Within this pathway, load may be progressed, maintained, reduced, or rehabilitation may be paused according to symptom stability, functional response, load tolerance, medication changes, and the presence of safety concerns. The framework also identifies circumstances requiring communication with the prescribing clinician or urgent medical assessment, such as concerning NSAID dosing or self-adjustment, potential contraindications or interactions, or suspected serious adverse medication events. By establishing NSAID use as a shared decision-making variable, DICE is proposed to facilitate more consistent communication among healthcare and performance professionals and to support more informed rehabilitation decisions (Figure 1).

**Figure 1:**
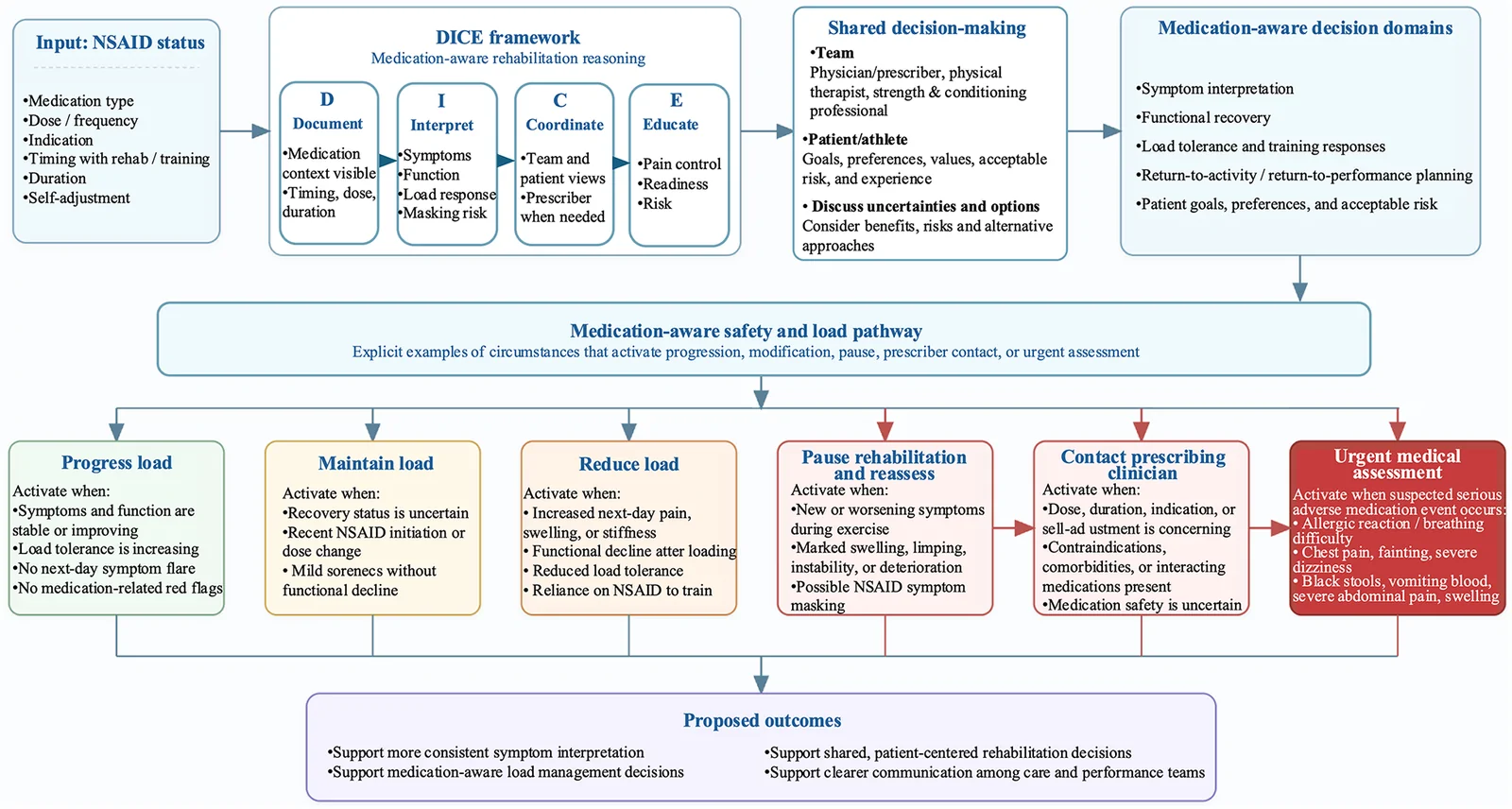
The DICE framework for medication-aware rehabilitation reasoning based on NSAID medication status. The framework comprises four sequential components: Document, Interpret, Coordinate, and Educate. Document captures relevant NSAID-related information, including medication type, dose, timing, duration, and indication. Interpret integrates medication context with symptom responses, functional capacity, and load tolerance. Coordinate facilitates information sharing among patients, rehabilitation professionals, and prescribing clinicians. Educate supports patient understanding of symptom control, recovery uncertainty, and medication-related considerations. These components inform shared decision-making and a medication-aware safety and load pathway, including when to progress, maintain, reduce, or pause rehabilitation exposure, when to contact the prescribing clinician, and when urgent medical assessment is required. The outcomes are presented as proposed or intended outcomes. They represent the DICE framework's intended support for symptom interpretation, medication-aware load management, clearer communication among care and performance teams, and rehabilitation-centered shared decision-making for patients, athletes, or individuals undergoing rehabilitation.

## 7 Clinical application of the DICE framework: an illustrative vignette

To illustrate how DICE may support rehabilitation decision-making, the following vignette provides a conceptual example of how medication status can be integrated into clinical reasoning. This example is intended to demonstrate the application process of DICE rather than represent a validated clinical protocol or specific treatment recommendation.

A 24-year-old professional soccer player presents with a grade II hamstring strain sustained during training. The athlete aims to return to team training within three weeks because of an upcoming competition. A sports physician prescribes ibuprofen (400 mg, twice daily) for five days to manage pain during the early rehabilitation phase. The athlete reports taking the morning dose approximately 60 min before the rehabilitation session and the second daily dose approximately 12 h later because the medication reduces discomfort and allows completion of prescribed exercises. The medication is documented by the rehabilitation team, including the indication, dose, timing, duration, and whether use was prescribed or self-initiated.

Document. The rehabilitation team records NSAID-related information alongside injury characteristics, current rehabilitation stage, and loading exposure. During communication, the athlete reports that pain during exercise has decreased from 6/10 to 2/10 after NSAID use. However, the team also documents that symptom changes occur under pharmacological pain modulation and therefore should not be interpreted independently as evidence of recovered tissue capacity.

Interpret. The athlete completes the planned rehabilitation session with minimal discomfort and demonstrates improved movement quality during exercise. The immediate assessment is conducted approximately 60 min after the morning ibuprofen dose, meaning that symptom and movement responses are assessed during a period of medication exposure. Accordingly, the immediate reduction in pain is interpreted as improved symptom control in the context of medication exposure. Approximately 24 h after the rehabilitation session, which was approximately 12 h after the second daily dose, the athlete reports increased posterior thigh stiffness and mild soreness. Functional assessment shows improved walking tolerance but eccentric hamstring strength remains approximately 15% lower than the contralateral side. These delayed symptoms and functional findings are considered alongside medication timing rather than interpreted in isolation. For this conceptual vignette, the combination of increased 24-hour symptoms and a defined eccentric strength deficit is interpreted as evidence that load tolerance may not yet have fully recovered. These findings are illustrative rather than validated progression criteria; they are intended to demonstrate how medication context, delayed symptom responses, and functional deficits could be integrated into clinical reasoning.

Coordinate. The physical therapist discusses delayed symptoms and functional findings with the strength and conditioning professional and sports physician. Because the athlete has a strong desire to return for competition, the team considers both the athlete's goals and the potential risk of premature progression. The prescriber is contacted to review whether continued NSAID use remains appropriate and whether medication timing may influence interpretation of rehabilitation responses.

Educate. The athlete is informed that reduced pain during NSAID use may facilitate participation but does not necessarily indicate full recovery of tissue capacity. The team explains the importance of reporting delayed symptoms, medication timing, and changes in function to support shared decision-making.

Shared decision-making. Rather than progressing training solely based on reduced pain during the session, the team decides to maintain the current rehabilitation load for several days because the athlete reports increased stiffness and soreness at approximately 24 h after loading and demonstrates an approximately 15% eccentric hamstring strength deficit compared with the contralateral side. The team continues to monitor 24-hour symptom responses and reassesses eccentric strength and functional performance before further progression. For the purposes of this conceptual example, progression would be considered when the athlete demonstrates stable or improving 24-hour symptom responses together with improved eccentric strength and functional performance across repeated exposures, without clinically meaningful deterioration after loading. These criteria are illustrative and have not been empirically validated as DICE-specific progression thresholds. This example demonstrates how DICE may alter clinical reasoning by shifting decision-making from immediate symptom relief toward an integrated assessment of medication context, functional recovery, and repeated load tolerance. To facilitate practical implementation of the DICE framework, a medication-aware documentation checklist is provided (Table 2). This checklist summarizes key information that may support consistent communication and decision-making among rehabilitation professionals, prescribers, and patients, athletes, or individuals undergoing rehabilitation.

**Table 2:** Medication-aware documentation checklist based on the DICE framework.

DICE component	Documentation item	Clinical purpose
Document	NSAID name/class	Identify medication context influencing symptom interpretation
Document	Dose and frequency	Consider potential magnitude of symptom modulation
Document	Timing relative to rehabilitation or training	Determine whether symptoms are assessed under medication effect
Document	Duration of use	Consider short-term versus prolonged exposure
Document	Indication for use	Clarify whether medication is prescribed for injury management or other reasons
Document	Prescribed or self-initiated use	Identify need for medication-related discussion or education
Interpret	Immediate pain response during activity	Assess symptom control while considering medication influence
Interpret	Delayed symptoms (hours after activity/next day)	Evaluate recovery response beyond immediate analgesic effects
Interpret	Swelling, stiffness, range of motion, strength, movement quality	Assess objective indicators of functional recovery
Coordinate	Communication among physician, physical therapist, and strength and conditioning professional	Ensure shared interpretation of medication and rehabilitation status
Coordinate	Need for prescriber review	Address medication safety or adjustment considerations when needed
Educate	Understanding of symptom relief versus recovery	Support informed participation in rehabilitation decisions
Decision	Progress, maintain, or modify load	Integrate medication status with symptoms, function, and load tolerance

Footnote: This checklist is intended as an illustrative decision-support tool to facilitate medication-aware rehabilitation reasoning. It is not an empirically validated assessment instrument or a substitute for clinical judgment. Application should be individualized according to tissue characteristics, stage of healing, NSAID class, dose, timing, duration of use, and patient-specific goals and preferences.

## 8 Limitations and future directions

The DICE framework is proposed as a conceptual model to integrate NSAID-related information into rehabilitation decision-making. Although it is based on established principles of symptom monitoring, load management, and collaborative care, its clinical effectiveness and implementation feasibility require further empirical validation. At present, evidence regarding its reliability, acceptability, interdisciplinary uptake, and impact on rehabilitation outcomes is lacking.

In addition, the applicability of DICE across different musculoskeletal conditions, healthcare systems, and sport settings remains to be determined. Future research should evaluate its feasibility in clinical practice and examine whether its implementation influences decision consistency, medication-related safety considerations, rehabilitation progression, and return-to-activity outcomes. Prospective observational studies and implementation research will be important to refine the framework and determine its potential value in real-world rehabilitation settings.

## 9 Conclusion: making NSAID Use visible in rehabilitation decision-making

NSAID use should not remain merely background information in the medical history. Instead, it should become a practical variable in symptom interpretation, load progression, and shared decision-making. This article does not argue that physical therapists or strength and conditioning professionals should assume responsibility for medication prescribing. Rather, it emphasizes that medication use should be incorporated into rehabilitation reasoning. Through documentation, interpretation, coordination, and education, the team can more clearly distinguish among pain control, functional recovery, and load tolerance. This distinction can support more consistent, transparent, and rehabilitation-centered decision-making for patients, athletes, or individuals undergoing rehabilitation. Rather than replacing existing pain-monitoring, load-management, or return-to-sport frameworks, DICE complements these approaches by explicitly incorporating medication status into rehabilitation reasoning and shared decision-making. Therefore, effective shared decision-making requires not only interprofessional coordination but also meaningful patient involvement, transparent communication of uncertainty, and recognition of individual goals and acceptable levels of risk.

## Data Availability

The original contributions presented in the study are included in the article/Supplementary Material, further inquiries can be directed to the corresponding author.
